# Can racial disparities in optimal gout treatment be reduced? evidence from a randomized trial

**DOI:** 10.1186/1741-7015-10-15

**Published:** 2012-02-09

**Authors:** Jasvinder A Singh

**Affiliations:** 1 Medicine Service, Birmingham VA Medical Center and Department of Medicine, University of Alabama, Birmingham, AL, USA; 2 Center for Surgical Medical Acute care Research and Transitions (C-SMART), Birmingham VA Medical Center, Birmingham, AL, USA; 3 Division of Epidemiology, School of Public Health, University of Alabama, Birmingham, AL, USA; 4 Department of Orthopedic Surgery, Mayo Clinic School of Medicine, Rochester, MN, USA; 5 University of Alabama, Faculty Office Tower 805B, 510 20th Street S, Birmingham, AL 35294, USA

**Keywords:** Gout, Disparity, Race, treatment, Febuxostat, Allopurinol, randomized, African-American

## Abstract

There is a disproportionate burden of gout in African-Americans in the U.S. due to a higher disease prevalence and lower likelihood of receiving urate-lowering therapy (ULT), compared to Caucasians. There is an absence of strong data as to whether the response to ULT differs by race/ethnicity. *BMC Musculoskeletal Disorders *recently published a secondary analyses of the CONFIRMS trial, a large randomized controlled, double-blind trial of 2,269 gout patients. The authors reported that the likelihood of achieving the primary study efficacy end-point of achieving serum urate < 6 mg/dl was similar between African-Americans and Caucasians, for all three treatment arms (Febuxostat 40 mg and 80 mg and allopurinol 300/200 mg). More importantly, rates were similar in subgroups of patients with mild or moderate renal insufficiency. Adverse event rates were similar, as were the rates of gout flares. These findings constitute a convincing evidence to pursue aggressive ULT in gout patients, regardless of race/ethnicity. This approach will likely help to narrow the documented racial disparities in gout care.

Please see related article: http://www.biomedcentral.com/1471-2474/13/15

## Background

Gout is the most common inflammatory arthritis in the U.S. that affects 4% of the general U.S. population [1]. The prevalence of gout is increasing in the U.S. [1-3], related at least partially to a rising rates of obesity and hypertension [1]. Based on the National Health and Nutrition Examination Survey (NHANES) 2007-2008, it is estimated that 6 million Caucasians and 1.2 million African-Americans in the U.S. have gout [1]. Gout presents with an extremely painful intermittent inflammatory arthritis, which over time, progresses to a chronic, deforming arthritis similar to rheumatoid arthritis. In addition to causing musculoskeletal morbidity and urate kidney stones, gout is an independent risk factor for cardiovascular morbidity and mortality [4-7].

The treatment of gout includes two approaches, treatment of acute episodes with medications that are anti-inflammatory including non-steroidal anti-inflammatory drugs (NSAIDs), colchicine or corticosteroids (oral, systemic or intra-articular) and the long-term treatment of hyperuricemia with medications that either reduce the production of uric acid (urate-lowering therapy (ULT) with xanthine oxidase inhibitors, allopurinol or febuxostat) or increase the excretion of urate (uricosurics such as probenecid [available in U.S.]; benzbromarone and sulfinpyrazone [not available in U.S.]) [8]. Due to a higher efficacy regardless of urate overproducer versus underexcretor status, allopurinol or febuxostat is used far more commonly than uricosurics. Despite availability of efficacious treatments, less than 50% patients treated with allopurinol, the commonest ULT, achieve the target serum urate < 6 mg/dl [9,10] that is associated with lower risk of gout flares, tophi and medical care costs [9,11-15].

### Racial Disparities in Gout Prevalence and Optimal Treatment

African-Americans have a higher prevalence of gout compared to Caucasians, ranging from 2-fold higher in an earlier study [16] to 1.25-fold higher in a recent analyses of NHANES 2007-08 [1]. An analysis of the 2002 US National Ambulatory Medical Care Survey and National Hospital Ambulatory Medical Care Survey found that African-Americans with gout were less likely to receive ULT compared to Caucasians [17]. The Institute of Medicine has identified that racial disparities in health care are common and that they are unacceptable [18]. Specifically, African Americans and Hispanics tend to receive lower quality of healthcare across a range of chronic conditions, and these disparities are found across a range of clinical settings, such as public and private hospitals. The report suggested multifaceted approach to reducing and eliminating these disparities, including raising public and health care professionals' awareness of the problem, health system interventions such as following published guidelines and improving health care access, educational tools for patients to improve participation in their care and decision-making and policy and regulatory strategies. The higher prevalence of gout coupled with lower likelihood of receiving ULT leads to a disproportionate morbidity of gout in African-Americans in the U.S. It is not known to what extent underutilization of ULT in African-Americans with gout is due to socioeconomic factors, health care insurance and access, distance to the nearest health care facility, patient preference, health literacy or health care disparity.

### CONFIRMS trial and Implications of Race/Ethnicity Analyses

CONFIRMS was a Phase 3, double-blind randomized controlled trial (RCT) that examined the comparative efficacy and safety of febuxostat in 2,269 subjects, who were randomized in 1:1:1 ratio to a daily dose of febuxostat 40 mg, febuxostat 80 mg, or allopurinol 300 mg (200 mg in patients with moderate renal impairment) [13]. In a recent article published in *BMC Musculoskeletal Disorders*, Wells et al., conducted a secondary analysis of the CONFIRMS trial, analyzing the efficacy and safety outcomes for African-Americans (10%) versus Caucasians (82%) enrolled in the trial [19]. They reported that febuxostat at both doses as well as allopurinol at the 300/200 mg dose was equally efficacious in achieving target serum urate < 6 mg/dl in both African-Americans as compared to Caucasians, as well as in subgroups of patients with mild renal insufficiency or moderate renal insufficiency. The risk of gout flares and the safety profile also seemed comparable between African-Americans and Caucasians, i.e., similar proportions reported at least one adverse event. Febuxostat 80 mg dose was more efficacious than 40 mg dose and allopurinol 300/200 mg dose. However, the low dose of allopurinol used in this study is effective in achieving target serum urate < 50% gout patients [20], and is considered suboptimal as per the current state-of-the-art gout treatment, where titration to the maximum approved dose of 800 mg in the U.S. is recommended, since the rate of allopurinol hypersensitivity seems to be independent of allopurinol dose used [21], contrary to what was previously suspected. An interesting observation, not discussed by authors, was that a lower proportion of African-Americans had liver function test abnormality (ALT elevation 2-times or higher, 2% vs. 13%, respectively) compared to Caucasians; however, these data were provided as an aggregate and not by treatment group and the rate of AST elevations seemed similar. Another observation was that febuxostat 40 mg was associated with significantly lower rates of achieving target serum urate < 6 mg/dl in African-Americans than Caucasians (38% vs. 55%, p = 0.046). The authors attribute this, at least partially, to lower compliance with Febuxostat 40 mg in African-Americans compared to Caucasians (66% vs. 82%), greater for this treatment arm than the other two (febuxostat 80 mg and allopurinol).

This is the first large randomized gout trial, for which separate analyses have been performed by race. It is reassuring to see that the chances of successful treatment with ULT are not different between African-Americans and Caucasians. These findings have significant implications. They support the notion that if treated optimally, both African-Americans and Caucasians with gout respond similarly to ULT. More importantly, the safety seemed similar in African-Americans and Caucasians with gout, and a possibility of lower risk of liver toxicity in African-Americans, which should be investigated further in future studies. The results of this study provide at least one way to reduce racial disparities in heath care of patients with gout, i.e., by increasing awareness of the public and providers' that patients' race/ethnicity does not impact the efficacy and safety of ULT in gout. Evidence-based gout quality indicators published 7 years ago emphasize the importance of achieving target serum urate < 6 mg/dl and using ULT as the main treatment strategy in gout [22].

What is known about racial disparities and their elimination in other musculoskeletal disorders? Racial [23,24] disparities in joint arthroplasty have been demonstrated with African Americans and Hispanics with lower utilization rates of knee and hip arthroplasty. A recent study also indicates smaller gains in well-being and pain/functional improvements after total joint arthroplasty in African Americans compared to Caucasians [25]. Fewer black patients (16%) with rheumatoid arthritis preferred aggressive treatment compared with white patients (51%) [26]. To our knowledge, very limited literature exists regarding how to best eliminate these disparities. It seems likely that increasing patient awareness of gout and its effective treatments and institution of aggressive treatment of gout in African Americans are two strategies that are likely to be most helpful first steps in addressing health disparities in gout.

### Conclusions: What is the take home message?

This study provides the evidence for the first time that ULT in gout patients is equi-efficacious in African-Americans and Caucasians in a randomized trial. The caveats of healthier population effect, Hawthorne effect, generalization of findings from group level to individuals, and differences in health behaviors between patients in clinical trials versus office practices that exist in translating findings from every randomized trial to clinical practice, exist in this case as well. Large sample pharmacovigilance studies are needed to truly assess differences in safety of ULT by race. Despite these limitations, this study contributes significant new knowledge in the area of comparative efficacy and safety of ULT in gout between African-Americans and Caucasians. While racial disparities in health care have complex etiology, they are unacceptable. These data should encourage physicians to aggressively treat both African-Americans and Caucasians with gout.

## Competing interests

There are no financial conflicts related to this work. JAS has received investigator-initiated research grants from Takeda and Savient; consultant fees from Takeda, Ardea, Savient, Allergan, Novartis and URL pharmaceuticals; and is a member of the executive of OMERACT, an organization that develops outcome measures in rheumatology and receives arms-length funding from 36 companies.

## Authors' contributions

JAS: Concept of the editorial, literature review, preparation of editorial and revision

## Authors information

JAS is a rheumatologist-epidemiologist, an Associate Professor of Medicine and Epidemiology at the University of Alabama at Birmingham and staff physician at the Birmingham Veterans Affairs Medical Center. JAS is a member of the American College of Rheumatology and serves as a committee member on the Guidelines subcommittee of the American College of Rheumatology's Quality of Care committee, Veterans Affairs Rheumatology Field Advisory Committee and the executive committee of the Outcome Measures in Rheumatology Trials (OMERACT).

## Pre-publication history

The pre-publication history for this paper can be accessed here:

http://www.biomedcentral.com/1741-7015/10/15/prepub
